# Supplementary material for: Content-rich biological network constructed by mining PubMed abstracts
Source: BMC Bioinformatics. 2004 Oct 8;5:147. doi: 10.1186/1471-2105-5-147 (PMC528731; doi:10.1186/1471-2105-5-147)
Supplement: Additional File 2 — The original results of the above study (non-essential files are deleted to keep the file size under the limit set by BMC bioinformatics). [file 1471-2105-5-147-S2.bz2 › chilibotAdditionalFile2/dip05/512ID9565586E10814/html/AR_BAG1.html]

 


 **AR** and **BAG1** 
  
Found 14 abstracts in PubMed, retrieved 05.  
 

 What does Google say? 
 PDF only 
| .edu only 

---

**Interactive relationship** (e.g. stimulation, inhibition, etc)

**Stimulatory relationship**- Although Bag1L, Bag1M, and  **Bag1**  can bind the androgen receptor  [ **AR** ]   **AR**  in vitro, only Bag1L enhanced  **AR**  transcriptional activity.  Ref: 11278763 J Biol Chem, 2001
**Neutral relationship**- Forced nuclear targeting of Bag1M, but not  **Bag1**  or Bag1S, resulted in potent  **AR**  coactivation, indicating that Bag1M possesses the necessary structural features provided it is expressed within the nucleus.  Ref: 11278763 J Biol Chem, 2001

**Non-interactive relationship** (e.g. studied together, co-existance, homology, etc.)

- Based on repeated isolations, high field infection rates and abundance, Culex appeared to be the vectors of BAG  [ **BAG1** ] , BBK,  **Ar**  D 65239 BAG  [ **BAG1** ]  like, and WN viruses, Ae.  Ref: 11476327 J Med Entomol, 2001
- ochraceus of RVF virus, Mansonia of WN and BAG  [ **BAG1** ]  viruses, Mimomyia of WN and BAG  [ **BAG1** ]  viruses, and Phlebotomine of SAB, CHP,  **Ar**  D 95537, and GF viruses.  Ref: 11476327 J Med Entomol, 2001
- six Flaviviruses, Bagaza BAG  [ **BAG1** ] ,  **Ar**  D 65239, Wesselsbron WSL, West Nile WN, Koutango KOU, Saboya SAB.  Ref: 11476327 J Med Entomol, 2001
